# A 12-month, prospective, observational study of ranibizumab in treatment-naïve Taiwanese patients with neovascular age-related macular degeneration: the RACER study

**DOI:** 10.1186/s12886-020-01715-3

**Published:** 2020-11-25

**Authors:** Wen-Chuan Wu, Jiann-Torng Chen, Ching-Yao Tsai, Chien-Liang Wu, Cheng-Kuo Cheng, Yun-Dun Shen, Arslan Tsai, Pei-Chang Wu

**Affiliations:** 1 Kaohsiung Medical University Chung-Ho Memorial Hospital, Sanmin District, Kaohsiung City, Taiwan; 2 Tri-Service General Hospital, National Defense Medical Centre, Chenggong Road, Taipei City, Taiwan; 3 Taipei City Hospital, Zhengzhou Rd, Taipei City, Taiwan; 4 Municipal Wan Fang Hospital, Xinglong Road, Taipei City, Taiwan; 5 Shin Kong Wu Ho-Su Memorial Hospital, Shilin District, Taipei City, Taiwan; 6 Taipei Medical University-Shung Ho Hospital, Zhonghe District, New Taipei City, Taiwan; 7 Clinical Development and Medical Affairs, Novartis Taiwan, Taipei City, Taiwan; 8 Department of Ophthalmology, Kaohsiung Chang Gung Memorial Hospital, Dapi Road, Niaosong District, Kaohsiung City, Taiwan

**Keywords:** nAMD, Observational, Ranibizumab, Real-world, Taiwan, Treatment-naïve

## Abstract

**Background:**

The current National Health Insurance scheme in Taiwan reimburses 3 initial plus 4 additional injections of ranibizumab 0.5 mg for eligible patients with neovascular age-related macular degeneration (nAMD). The Ranibizumab AMD Clinical Efficacy in Real-world practice (RACER) study aimed to observe the effectiveness of ranibizumab injections under this reimbursement system.

**Methods:**

RACER was a 12-month, prospective, observational study conducted in treatment-naïve, adult Taiwanese patients with nAMD. Patients received intravitreal ranibizumab 0.5 mg injections in adherence with local prescribing information.

**Results:**

Of 161 patients enrolled, 114 (70.8%) completed the 12-month study. Overall, patients received a mean (standard deviation [SD]) of 4.3 (1.7) ranibizumab injections. The mean (SD, [95% confidence interval], *P* value) gain in best-corrected visual acuity (BCVA) from baseline at Month 3 was 5.2 (12.2, [3.1, 7.3] letters, *P* < 0.0001) and at Month 12 was 3.4 (15.4, [0.2–6.6] letters, *P* = 0.0352). Mean central retinal thickness also decreased from baseline at Months 3 and 12 (both *P* < 0.001). In subgroup analyses, better treatment outcomes at Months 3 and 12 were observed among patients who received a loading dose and those who had a shorter duration of nAMD at baseline. Adverse events were reported in 58.4% of patients; most (94.4%) were mild-to-moderate in severity and 98.8% were deemed unrelated to study treatment.

**Conclusions:**

Treatment with ranibizumab 0.5 mg resulted in significant improvements in visual outcomes among treatment-naïve Taiwanese patients with nAMD. Early treatment and frequent dosing in the real-world setting may be the key to achieving better outcomes.

**Supplementary Information:**

The online version contains supplementary material available at 10.1186/s12886-020-01715-3.

## Key findings

Treatment with ranibizumab under the current National Health Insurance reimbursement scheme of 3 initial plus 4 additional injections resulted in significant improvements in visual outcomes in treatment-naïve Taiwanese patients with neovascular age-related macular degeneration.

## Background

Neovascular age-related macular degeneration (nAMD) is a rapidly progressing disease which impacts central vision; visual loss becomes irreversible if diagnosis and treatment are delayed. Since the receipt of Food & Drug Administration approval in 2006 [1] as the first anti-vascular endothelial growth factor (VEGF) treatment for nAMD based on results from the MARINA and ANCHOR trials [2, 3] ranibizumab has been approved for the treatment of nAMD in many countries [4]. However, treatment patterns for nAMD are subject to country-specific reimbursement systems, which have changed and evolved over time based on real-life conditions.

In Taiwan, the prevalence of nAMD is 7.3% in people aged ≥65 years and 11.4% in those aged ≥80 years [4, 5]. Ranibizumab was approved for the treatment of nAMD in Taiwan in 2009 [4]. Since 2011, the Taiwan National Health Insurance (NHI) reimbursed limited injections (up to 3 doses within 1 year) for nAMD, subject to an application process that needed to be pre-approved by reviewers [4]. Effective from August 2014, the reimbursement was relaxed from 3 injections to 3 + 4 injections for 2 rounds of application within 1 year, if eligibility criteria are met. The effectiveness of ranibizumab under real-world conditions remains a worthy source of evidence to demonstrate how relaxation of reimbursement has an impact on patient outcomes and if there are any implications with regard to simplification and acceleration of the pre-approval process.

The **R**anibizumab **A**MD **C**linical **E**fficacy in **R**eal-world practice (RACER) study was designed to observe the real-world effectiveness and safety of ranibizumab over a 12-month period in treatment-naïve patients with nAMD who were eligible for the new reimbursement scheme.

## Methods

### Study design

RACER was an open-label, prospective, observational study conducted at 7 centres in Taiwan from May 2014 to May 2017.

Eligible patients were treated with ranibizumab according to the approved labeling dosage and administration: once a month for 3 consecutive months with regular follow-up of visual acuity (VA) and disease reactivation, thereafter, with a predominantly PRN retreatment regimen. There is a no switch in anti-VEGF policy under the NHI reimbursement criteria. Criteria for reimbursement eligibility are listed in Table S1.

The study did not interfere with any diagnostic or therapeutic measures taken by the treating physicians. Data were collected at baseline (Day 1), and Months 3 and 12.

### Study population

Treatment-naive, adult Taiwanese patients with visual impairment due to nAMD, for whom intravitreal treatment with ranibizumab 0.5 mg was prescribed in the course of routine medical practice were included. Patients were excluded if they had concomitant conditions in the study eye that could prevent the improvement in VA on study treatment in the investigator’s opinion; received systemic treatment with any VEGF inhibitor in the 30 days prior to enrolment; or had active, or history of, ocular inflammation or infection within 30 days prior to screening. Pregnant or nursing women were also excluded.

The study protocol and amendments were approved by an independent ethics committee or institutional review board, as appropriate, for each site. The study was conducted in compliance with the Declaration of Helsinki, and in accordance with Good Clinical Practices and applicable regulatory requirements. Patients provided written informed consent before enrollment.

### Study endpoints and assessments

The primary objective of the study was to evaluate the effectiveness of ranibizumab 0.5 mg with respect to mean change in BCVA from baseline at Month 3. Key secondary endpoints included mean change in BCVA and central retinal thickness (CRT) at Month 12, treatment exposure to ranibizumab 0.5 mg, and safety events over 12 months. Post hoc analyses included the mean change in BCVA and CRT categorised by (a) number of loading doses (patients who received ≥3 ranibizumab injections in the first 3 months [loading group] vs patients who received < 3 injections in the first 3 months [non-loading group]), (b) number of ranibizumab injections (> 3 vs ≤3), and (c) duration of nAMD at baseline (< 3 months vs ≥ 3 months).

BCVA was assessed using an Early Treatment Diabetic Retinopathy Study (ETDRS) protocol at an initial distance of 4 m. Optical coherence tomography (OCT) was performed at the discretion of the physician following local standard medical practice. Fluorescein angiography was conducted in conjunction with colour fundus photography on both eyes at screening and in the study eye during scheduled visits, if necessary. Safety evaluation consisted of collecting adverse events (AEs) and serious AEs (SAEs) over the 12-month observational period.

### Statistical analyses

Assuming that mean (SD) change from baseline in BCVA letter score at Month 3 was 7.0 (8.0) letters, a sample size of 160 patients provided 80% power to test the superiority with a 20% dropout rate. The primary endpoint was tested using a one-sided superiority test at a significance level of 0.025 to detect a 5-letter improvement from baseline in BCVA score at Month 3; based on results from previous trials [2, 3]. A pre-specified exploratory analyses planned before database lock included tests for significance of change from baseline at a 2-sided significance level of 0.05, which was used in a recent real life report [6]. The difference of continuous variables change from baseline was compared using a paired T test. If the data had not been well-modelled by a normal distribution, the Wilcoxon Signed-rank test was used. All other efficacy endpoints and safety were summarised descriptively. Observed data were used without any imputation. All analyses were performed by the Formosa Contract Research Organisation (Taipei, Taiwan).

The intent-to-treat (ITT) and safety population consisted of all patients who had baseline assessment, received ≥1 dose of ranibizumab and had ≥1 post-baseline assessment.

## Results

### Patient disposition and baseline characteristics

A total of 161 patients were enrolled, of whom 153 (95.0%) and 114 (70.8%) completed 3 and 12 months of study observation, respectively. The reasons for discontinuation were withdrawal of consent (40 [85.1%]), loss to follow-up (5 [10.6%]), death (1 [2.1%]), and other reasons (1 [2.1%]).

In the ITT population (*n* = 152), the mean age was 71.6 years, most patients were male (64.5%), and the mean duration of nAMD was 5.5 months (Table 1). About half of the population had haemorrhage and subretinal fibrosis (SRF), about a third had pigment epithelial detachment, and 16% had scar. Occult or minimally classic types of choroidal neovascularisation were twice as prevalent as the classic type (63.1% vs 36.9%, respectively). Indocyanine green angiography was performed in 51 patients, of whom 13 (25.5%) were diagnosed with polypoidal choroidal vasculopathy.

**Table 1:** Baseline demographics, ocular and disease characteristics for patients with nAMD treated with ranibizumab (ITT Population^a^)

Characteristics	ITT Population (*n* = 152)
Mean (SD) age, years	71.6 (10.8)
Gender, male, n (%)	98 (64.5)
Mean (SD) nAMD duration, months	5.5 (17.3)
Proportion of patients with nAMD duration ≤3 months, %	82.9
Mean (SD) BCVA, letters	47.5 (20.0)
Mean (SD) CRT, μm	385 (131.9)
**Color fundus photography, n (%)**	
Haemorrhage	87 (58.0)
PED	55 (36.7)
SRF	77 (51.3)
Scar	24 (16.0)
**CNV type, n (%)**	141 (98.6)
100% classic/predominantly classic	11 (7.8)/ 41 (29.1)
Minimally classic/occult with no classic component	55 (39.0)/ 34 (24.1)

*BCVA* best-corrected visual acuity, *CNV* choroidal neovascularisation, *CRT* central retinal thickness, *ITT* intent-to-treat, *n* number of patients, *nAMD* neovascular age-related macular degeneration, *SD* standard deviation

^a^Defined as patients who had baseline assessment, received at least 1 dose of observational drug and had at least 1 post-baseline assessment of the effectiveness variables. Data for BCVA and CRT was not available for 1 patient

### Efficacy

#### Overall

**Fig. 1:**
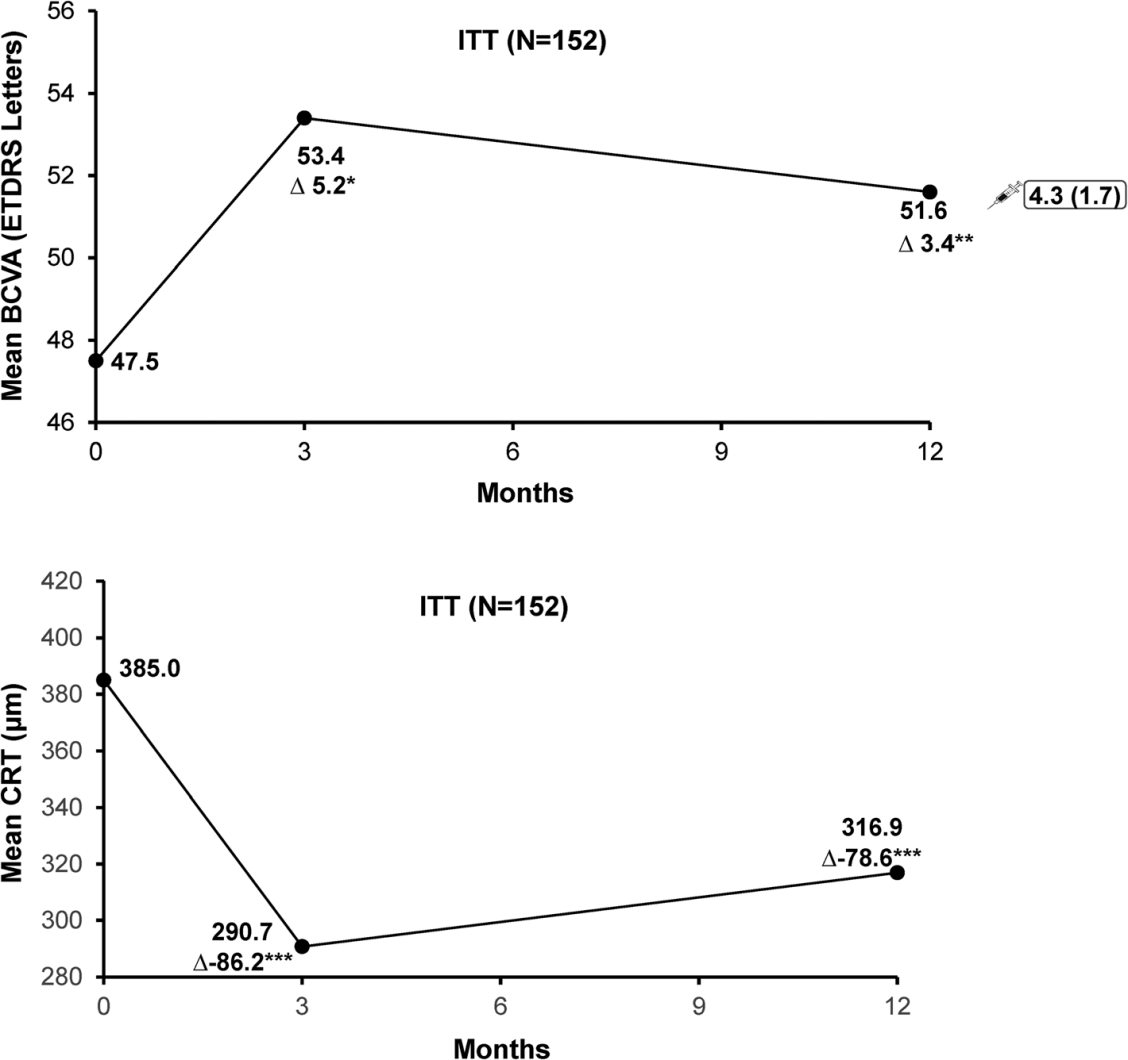
Mean change from baseline in BCVA and CRT at Months 3 and 12. *P < 0.0001; **P = 0.0352; ***P < 0.001. *P* values for BCVA based on a prescpecified exploratory analysis. ∆ represents mean gain in BCVA or mean reduction in CRT.  indicates mean number of injections. BCVA: baseline, *n* = 151; Month 3, *n* = 135; Month 12, *n* = 93. CRT: Baseline, *n* = 151; Month 3, *n* = 130; Month 12, *n* = 85**.** BCVA, best-corrected visual acuity; CRT, central retinal thickness; ITT, intent-to-treat; n, number of patients

Patients treated with ranibizumab showed a mean (SD, [95% confidence interval {CI}]) gain of 5.2 (12.2, [3.1, 7.3]) letters from baseline at Month 3. Although it did not satisfy the superiority test (*P* = 0.6676) due to a relatively limited number of injections in real-life as compared with controlled trials [2, 3], the exploratory analyses planned before database lock revealed a significant improvement from baseline at Month 3 (*P* < 0.0001); at Month 12 the mean gain in BCVA from baseline was 3.4 (15.4, [0.2–6.6]) letters, *P* = 0.0352. Mean CRT decreased from baseline at Months 3 and 12 (mean [SD] 86.2 [102.3] μm and 78.6 [97.5] μm, both *P* < 0.001, respectively, Fig. 1). For most symptoms except scar, ~ 90% of patients showed improvement or stable conditions over time (Table S2).

#### Subgroups

**Fig. 2:**
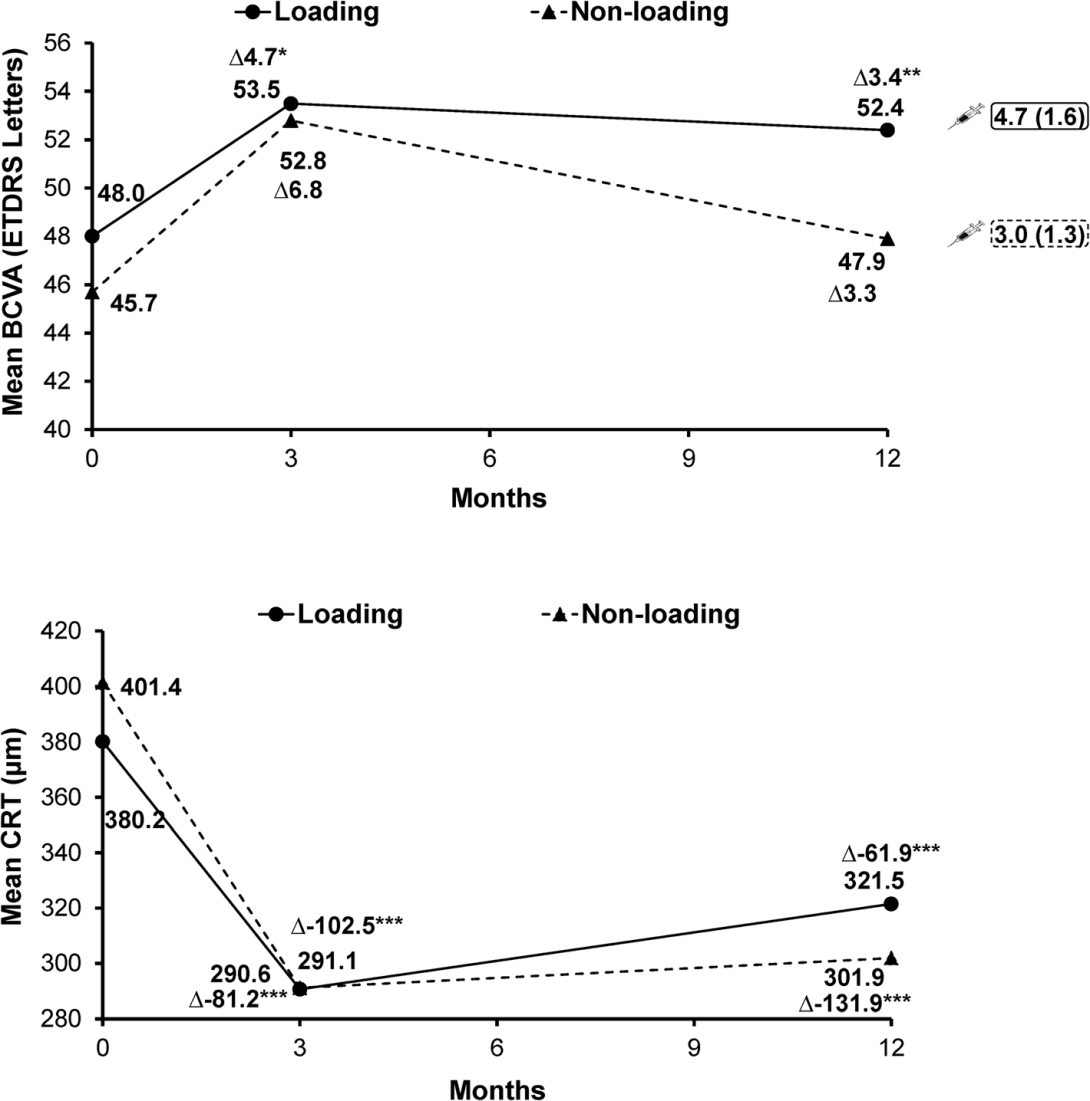
Mean change from baseline in BCVA and CRT at Months 3 and 12 by loading and non-loading dose subgroups**.** *P < 0.0001 (95% CI 2.8–6.7); **P = 0.0266; ***P < 0.001. ∆ represents mean gain in BCVA or mean reduction in CRT.  indicates mean number of injections. BCVA: baseline, loading *n* = 117, non-loading *n* = 34; Month 3, loading *n* = 106, non-loading *n* = 29; Month 12, loading *n* = 76, non-loading *n* = 17. CRT: baseline, loading *n* = 117, non-loading *n* = 34; Month 3, loading *n* = 100, non-loading *n* = 30; Month 12, loading *n* = 65, non-loading *n* = 20. BCVA, best-corrected visual acuity; CRT, central retinal thickness; ETDRS, Early Treatment for Diabetic Retinopathy Study; n, number of patients

Loading (*n* = 118; 77.6%) and non-loading (*n* = 34; 23.4%) dose subgroups were comparable for baseline demographic characteristics and nAMD duration (Table S3). The mean number of ranibizumab injections was higher in the loading (4.7 [1.6]) vs the non-loading dose subgroup (3.0 [1.3]). The proportion of patients who received 3 + 4 injections was 63.6% (75/118) in the loading dose subgroup vs 29.4% (10/34) in the non-loading dose subgroup. Notable improvements in BCVA from baseline at Months 3 and 12 were observed only in the loading dose subgroup (4.7 [10.1] and 3.4 [13.1] letters, *P* < 0.0001 and 0.0266, respectively, Fig. 2). At Month 12, notable and comparable reductions in mean CRT were observed in the loading vs non-loading dose subgroups (321.5 μm vs 301.9 μm, Fig. 2)**.**

**Fig. 3:**
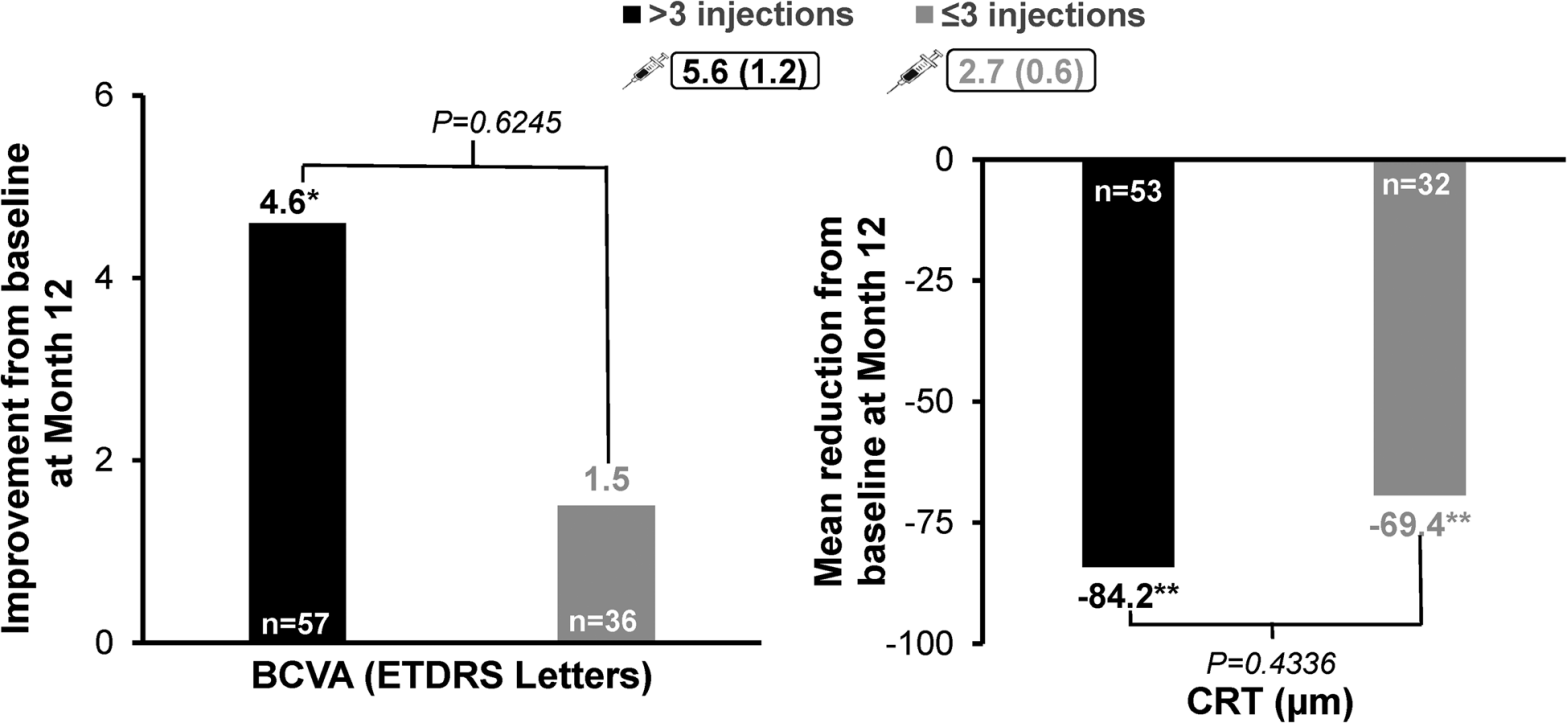
BCVA improvement and reduction in CRT from baseline at Month 12 by number of injections administered. *P = 0.0252;**P < 0.001.  indicates mean number of injections. BCVA: baseline, > 3 injections *n* = 85, ≤3 injections *n* = 66; Month 12, > 3 injections *n* = 57, ≤3 injections *n* = 36. CRT: baseline, > 3 injections *n* = 84, ≤3 injections *n* = 67; Month 12, > 3 injections *n* = 53, ≤3 injections *n* = 32. BCVA, best-corrected visual acuity; CRT, central retinal thickness; ETDRS, Early Treatment for Diabetic Retinopathy Study; n, number of patients; SD, standard deviation

Patients receiving ≤3 or > 3 ranibizumab injections were comparable in baseline demographic characteristics and nAMD duration (Table S3). Comparable mean gains in BCVA from baseline were observed in both subgroups at Month 3 (*P* = 0.0011, and *P* = 0.0007, respectively; between-group *P* = 0.2048); the gains were maintained at Month 12 in patients receiving > 3 injections (*P* = 0.0252, Fig. 3). The mean reduction in CRT from baseline at Month 12 was notable in both subgroups (Fig. 3).

Baseline demographic characteristics were generally comparable between patients with nAMD duration ≤3 months or > 3 months (Table S3). Mean [SD] ranibizumab injections were higher in patients with nAMD duration > 3 months vs ≤3 months (5.0 [1.7] vs 4.2 [1.7], between-group *P* = 0.0289).

In the loading dose subgroup a significant improvement in BCVA at Month 3 was seen only among patients with nAMD duration ≤3 months vs > 3 months (mean [SD, 95% CI], 5.1 [10.6, 2.9–7.4] vs 2.8 [7.1, − 0.8-6.3], *P* < 0.0001 and *P* = 0.1126, respectively, between-group *P* = 0.1720); the BCVA improvement at Month 12 was similar between the subgroups (mean [SD, 95% CI], 3.3 [13.9, − 0.3–7.0] vs 3.8 [10.5, − 1.5–9.0], *P* = 0.0759 and *P* = 0.1431, respectively, between-group *P* = 0.8999). Final CRT outcomes at Month 3 were significantly different between patients with nAMD duration ≤3 months vs > 3 months, in the overall population and in the loading dose subgroup (mean [SD], overall population: 282.6 [100.1] vs 328.6 [114.8], between-group *P* = 0.0264; loading dose subgroup: 278.1 [79.1] vs 344.0 [119.5], between-group *P* = 0.0094). The trend was maintained numerically at Month 12, although there was no significant between-group difference (mean [SD], overall population: 301.8 [88.6] vs 393.7 [194.9], between-group *P* = 0.0936; loading dose subgroup: 305.6 [86.8] vs 400.0 [211.1], between-group *P* = 0.2141).

Colour fundus photography results were generally comparable between all subgroups at baseline (Table S3) and after treatment (data not shown). However, no meaningful interpretations can be drawn from these data due to the limited number of cases graded by investigators across subgroups.

#### Treatment exposure

Overall, the mean (SD) number of ranibizumab injections received by patients in the ITT population was 4.3 (1.7). Over half of the population (55.9%, 85/152) was approved for the second round of treatment and received > 3 ranibizumab injections. The mean number of overall anti-VEGF injections received, including concomitant bevacizumab or aflibercept, was 4.5 (1.8).

### Safety

A total of 254 AEs were reported in 58.4% of patients. The majority of AEs (94.4%) were mild or moderate in severity. Allergic conjunctivitis was the ocular AE with the highest incidence (5.0%, Table 2), while upper respiratory tract infection was the non-ocular AE with the highest incidence (3.7%).

**Table 2:** Proportion of patients with AEs (Safety Population*)

Preferred term, n (%)	Safety Population (*N* = 161)*
**Number of patients with AEs, n (%)**	**94 (58.4)**
**Ocular AEs, n (%)**	**28 (17.4)**
Conjunctivitis allergic	8 (5.0)
Conjunctivitis	7 (4.3)
Cataract	6 (3.7)
Asthenopia	5 (3.1)
Conjunctival haemorrhage	5 (3.1)
Dry eye	5 (3.1)
Eye pain	5 (3.1)
Ocular discomfort	5 (3.1)
Lacrimation decreased	4 (2.5)
Retinal hemorrhage	4 (2.5)

*AE* adverse event, *N* total number of patients, *n* number of patients, *MedDRA* medical dictionary for regulatory activities, *SAE* serious AE

*Safety population consisted of all patients who received at least 1 dose of observational medication and had at least 1 post-baseline safety assessment. AEs ≥2% have been included. Preferred terms (MedDRA v2.0) are presented by descending order of frequency

Serious AEs (25 events) were reported in 11.8% of patients. Three SAEs from 2 patients were suspected to be treatment-related, but all of them resolved (1 case of coronary artery disease and 2 cases of vitreous haemorrhage [in the same patient]). There were no discontinuations related to treatment. One death was reported (due to autoimmune hepatitis) which was considered by the investigator to be unrelated to treatment.

## Discussion

In this prospective, observational study, ranibizumab treatment resulted in visual and anatomic improvements in treatment-naive Taiwanese patients with nAMD. Overall, patients in the RACER study showed a marked improvement in BCVA of 5.2 letters from baseline at Month 3 (*P* < 0.0001). Though the improvement did not reach the predefined criteria for superiority, which was expected to be achieved in naïve patients, it was maintained at 3.4 letters until Month 12 (*P* = 0.0352). The slight numerical drop in BCVA from Month 3 may be explained by the fact that patients were likely to have intensive treatment in the first 3 months, followed by a less intensive approach until Month 12 owing to the limited number of injections reimbursed. Nevertheless, the reduction in CRT from baseline was notable both at Months 3 and 12 (both *P* < 0.001) under the real-life treatment frequency. Results of this study are comparable with those from most real-world studies in other parts of the world. A meta-analysis of 12-month outcomes from 12 observational studies of naïve patients with nAMD reported mean (SD) VA gains of 3.5 (3.9) letters with a mean of 5.4 (0.7) injections [7].

Subgroup analysis by nAMD duration showed that among patients who received 3 loading doses, the BCVA and CRT outcomes at Month 3 were significantly improved only among patients with short disease duration (≤3 months). This finding is consistent with previous studies that have shown better outcomes with early treatment. Data from the UK Electronic Medical Records system showed significantly better VA among early-treated eyes and those with good VA (> 70 ETDRS letters) at baseline vs eyes that received delayed treatment and with lower VA at baseline [8]. Comparisons of 7-year outcomes between the study and fellow eyes of patients from the ANCHOR, MARINA, and HORIZON trials (SEVEN-UP) also revealed significantly better outcomes in the study vs fellow nAMD eyes, who later entered treatment during the study extension 2 years after the core study was completed [9]. These data emphasise the importance of early detection and treatment of nAMD.

Before relaxation of the NHI reimbursement in Taiwan, the report by Chang et al. (*N* = 229) on real-world use of ranibizumab 0.5 mg among Taiwanese patients showed that VA significantly improved among 51.8% of patients and stabilised in 38.3% of patients after 3 injections of ranibizumab [4]. Compared with other observational studies with a wide range of treatment frequencies from 4.8 to 7.0 injections/year [10–15], the 3 injections reimbursed in 1 year in Taiwan seemed low, and was later relaxed to 3 + 4 injections at the time of the observational period for RACER. Results from the RACER study are indicative of real-world clinical efficacy achieved with relaxation of NHI reimbursement in Taiwan. With more injections reimbursed, more frequent and intensive dosing of ranibizumab injections may delay the deterioration of naïve nAMD along the course of disease, as patients who received > 3 ranibizumab injections maintained their VA gains at Month 12 vs patients who received ≤3 injections.

Reimbursement schemes play an important role in determining outcomes, particularly in developing countries, where self-paying patients may not seek treatment until their symptoms significantly worsen, leading to potential under-treatment. In this study, over half of the study population were approved for the second round of application and received  > 3 injections, while other patients did not go for or were ineligible for the second application and received ≤3 injections. Further, a substantial delay in treatment administration was reported between the third and fourth injections (3.6 [2.3] months), an unnecessary prolongation due to the consecutive application process for the additional 4 injections, which may have influenced outcomes at 12 months [4]. Delays in treatment can also happen in other situations, such as seasonal holidays; patients who consistently skipped ranibizumab injections during holidays did worse in terms of BCVA gain (between-group *P* = 0.041) and exudation on OCT (between-group *P* = 0.007) than those who adhered to treatment [16].

Patients in the loading dose subgroup with significant BCVA improvement from baseline at Months 3 and 12 received a higher number of injections than those in the non-loading subgroup, which could be related to treatment selection by physicians. On the other hand, patients in the non-loading subgroup may have showed immediate yet transient therapeutic response to treatment, but being unable to follow-up regularly during the maintenance phase may have resulted in unmonitored recurrence, and consequently, worse BCVA outcomes at Month 12. Despite a numerical gain in mean BCVA at Month 12 (3.3 [23.5] letters, *P* = 0.5707) in the non-loading subgroup, the large SDs may be driven by patients who deteriorated with a drop in VA. While a limited number of injections may be sufficient for some patients with good response and stable disease, rigorous monitoring may still be required to detect disease recurrence as early as possible.

Most color fundus photography results were stable or improved over time; improvement was defined as change from presence at baseline to absence, and worsening was defined as change from absence at baseline to presence of symptoms. Improvement rates were most prominent in haemorrhage and SRF for 46.3 and 37.3% at Month 12 respectively. The worsening of scar is consistent with the course of disease; new scar rate at Month 12 was 17.9%, about double from 16% at baseline. (Table S2).

The majority of AEs (94.4%) were mild or moderate in severity and most AEs were unrelated to ranibizumab. These results were in line with previous studies, with no new safety concerns. The safety profile was consistent with the known safety profile of ranibizumab.

Potential strengths of the RACER study include a high external validity inherent to observational studies, which serves as a good reflection of current real-world practice under a reimbursement system that may influence treatment decision. It serves as an important benchmark to compare the current NHI reimbursement scheme to the original one, which only reimbursed 3 annual injections for nAMD, and to weigh the financial burden of 3 + 4 injections against the socioeconomic impact of sustainable BCVA benefits upon early and continuous treatment with a more simplified and accelerated pre-approval process.

Potential limitations include the small number of patients, physicians’ bias in treatment selection and consequently, treatment response. The study used subgroup analyses to identify associations between different subgroups. However, failure to specify subgroups of interest a priori may have led to imbalanced subpopulations; hence, results should be interpreted with caution. Finally, there is the challenge of missing data inherent to an observational study.

## Conclusions

In conclusion, results from the RACER study showed that treatment with ranibizumab 0.5 mg resulted in improved VA gains in treatment-naïve Taiwanese patients with nAMD. Frequent and intensive dosing delays the deterioration of naive nAMD. Early treatment and extended injections with regular follow-ups may be needed for ranibizumab-treated patients with nAMD in order to achieve better outcomes.

## Supplementary Information

**Additional file 1:** **Table S1.** Eligibility Criteria for NHI reimbursement [4]. **Table S2.** Colour fundus photography results compared with baseline (ITT population). **Table S3.**Baseline demographics, ocular and disease characteristics for patients with nAMD treated with ranibizumab 0.5 mg, by subgroups (ITT population).

## Data Availability

The datasets used and/or analysed during the current study are available from the corresponding author on reasonable request.

## Abbreviations

AE: Adverse event
BCVA: Best-corrected visual acuity
CI: Confidence interval
CRT: Central retinal thickness
ETDRS: Early treatment diabetic retinopathy study
ITT: Intent-to-treat
nAMD: Neovascular age-related macular degeneration
OCT: Optical coherence tomography
PED: Pigment epithelial detachment
PRN: Pro re nata
RACER: Ranibizumab AMD Clinical Efficacy in Real-world practice
SAE: Serious adverse event
SD: Standard deviation
SRF: Subretinal fibrosis
VEGF: Vascular endothelial growth factor
