# Supplementary material for: A 12-month, prospective, observational study of ranibizumab in treatment-naïve Taiwanese patients with neovascular age-related macular degeneration: the RACER study
Source: BMC Ophthalmol. 2020 Nov 25;20:462. doi: 10.1186/s12886-020-01715-3 (PMC7687747; doi:10.1186/s12886-020-01715-3)
Supplement: Supplementary file 1 — Additional file 1: Table S1. Eligibility Criteria for NHI reimbursement [4]. Table S2. Colour fundus photography results compared with baseline (ITT population). Table S3.Baseline demographics, ocular and disease characteristics for patients with nAMD treated with ranibizumab 0.5 mg, by subgroups (ITT population). [file 12886_2020_1715_MOESM1_ESM.docx]

**Table S1. Eligibility Criteria for NHI reimbursement(**[**4**](#_ENREF_4)**)**

| **Criteria** |  |
| --- | --- |
| Age | ≥50 years |
| FA and OCT | performed within the past month and compatible with a diagnosis of nAMD |
| BCVA | 0.05–0.5 (20/400–20/40) |

BCVA, best-corrected visual acuity; FA, fluorescein angiography; nAMD, neovascular age-related macular degeneration; OCT, optical coherence tomography

**Table S2**. **Colour fundus photography results compared with baseline (ITT population)**

|  | Improved, n (%) | No  change, n (%) | Worsened,  n (%) | *P* value† | Improved, n (%) | No change, n (%) | Worsened, n (%) | *P* value† |
| --- | --- | --- | --- | --- | --- | --- | --- | --- |
|  | **Month 3** | **Month 12** |  |  |  |  |  |  |
| Haemorrhage | 46 (42.2) | 61 (56.0) | 2 (1.8) | <0.001* | 31 (46.3) | 34 (50.7) | 2 (3.0) | <0.001* |
| PED | 20 (18.3) | 80 (73.4) | 9 (8.3) | 0.0614 | 8 (11.9) | 51 (76.1) | 8 (11.9) | >0.9999 |
| SRF (apparent) | 42 (38.5) | 61 (56.0) | 6 (5.5) | <0.001* | 25 (37.3) | 40 (59.7) | 2 (3.0) | <0.001* |
| Scar | 4 (3.7) | 92 (84.4) | 13 (11.9) | 0.0490* | 1 (1.5) | 54 (80.6) | 12 (17.9) | 0.0034* |

*Statistically significant, significance level=0.05
†The difference was compared by McNemar's test
ITT, intention-to-treat; n, number of patients; PED, pigment epithelial detachment; SRF, subretinal fibrosis

**Table S3**. **Baseline demographics, ocular and disease characteristics for patients with nAMD treated with ranibizumab 0.5 mg, by subgroups (ITT population)**

| Characteristics | Subcategories | | | | | | | | |
| --- | --- | --- | --- | --- | --- | --- | --- | --- | --- |
|  | **Loading (n=118)** | **No loading (n=34)** | ***P* value†** | **≤3 injections  (n=67)** | **>3 injections (n=85)** | ***P* value†** | **nAMD  ≤3 months (n=126)** | **nAMD  >3 months (n=26)** | ***P* value†** |
| Mean (SD) age, years | 70.1 (11.3) | 72.1 (9.1) | 0.7763 | 70.1 (11.3) | 72.9 (10.3) | 0.1203 | 70.9 (11.0) | 75.5 (9.1) | 0.0536 |
| Gender, male, n (%) | 77 (65.3) | 21 (61.8) | 0.7080 | 40 (59.7) | 58 (68.2) | 0.2751 | 81 (64.3) | 17 (65.4) | 0.9151 |
| Mean (SD) nAMD duration, months | 6.2 (19.1) | 3.1 (7.9) | 0.3982 | 5.5 (21.1) | 5.6 (13.6) | 0.7411 | 1.1 (0.6) | 27.2 (34.8) | <0.001* |
| Mean (SD) number of ranibizumab injections | 4.7 (1.6) | 3.0 (1.3) | <0.001* | 2.7 (0.6) | 5.6 (1.2) | <0.001* | 4.2 (1.7) | 5.0 (1.7) | 0.0289* |
| Mean (SD) BCVA, letters | 48 (20.1)^¶^ | 45.7 (19.8) | 0.5609 | 46.2 (20.9)^#^ | 48.5 (19.3) | 0.4838 | 49.1 (19.2)^§^ | 39.7 (22.1) | 0.0590 |
| Mean (SD) CRT, µm | 380.2 (131.4)^¶^ | 401.4 (134.3) | 0.1707 | 384.1  (132.1) | 385.7 (132.6)^^^ | 0.8987 | 379.5 (123.6)^§^ | 411.5 (166.7) | 0.8360 |
| Colour fundus photography^‡^ |  |  |  |  |  |  |  |  |  |
| Haemorrhage | 68 (57.6) | 19 (59.4) | 0.8590 | 38 (57.6) | 49 (58.3) | 0.9257 | 74 (59.7) | 13 (50.0) | 0.3633 |
| PED | 42 (35.6) | 13 (40.6) | 0.6004 | 20 (30.3) | 35 (41.7) | 0.1517 | 46 (37.1) | 9 (34.6) | 0.8113 |
| SRF (apparent) | 59 (50.0) | 18 (56.3) | 0.5304 | 29 (43.9) | 48 (57.1) | 0.1083 | 61 (49.2) | 16 (61.5) | 0.2522 |
| Scar | 16 (13.6) | 8 (25.0) | 0.1174 | 10 (15.2) | 14 (16.7) | 0.8016 | 19 (15.3) | 5 (19.2) | 0.5691 |

*Statistically significant
†The difference of continuous variables between treatment groups was compared by independent T test at a statistical significance level of 0.05. If the data had not been well-modelled by a normal distribution, the
Mann-Whitney U test would be used
‡The difference of categorical variables between treatment groups was compared by Chi-square test at a statistical significance level of 0.05. If expected number of any cell was less than 5, the Fisher's exact test was used
^¶^n=117; ^#^n=66^; ^^n=84; ^§^n=125
BCVA, best-corrected visual acuity; CNV, choroidal neovascularization; CRT, central retinal thickness;
ITT, intent-to-treat; n, number of patients; nAMD, neovascular age-related macular degeneration;
PED, pigment epithelial detachment; SD, standard deviation; SRF, subretinal fibrosis; VEGF, vascular endothelial growth factor
